# Trastuzumab increases the sensitivity of HER2-amplified human gastric cancer cells to oxaliplatin and cisplatin by affecting the expression of telomere-associated proteins

**DOI:** 10.3892/ol.2014.2793

**Published:** 2014-12-12

**Authors:** YONGPING LIU, YANG LING, QIUFENG QI, MING ZHU, MEIZHEN WAN, YAPING ZHANG, CHANGSONG ZHANG

**Affiliations:** 1 Clinical Oncology Laboratory, Changzhou Tumor Hospital Affiliated to Suzhou University, Changzhou, Jiangsu 213002, P.R. China; 2 Department of Oncology Medicine, Changzhou Tumor Hospital Affiliated to Suzhou University, Changzhou, Jiangsu 213002, P.R. China; 3 Department of Pathology, Changzhou Tumor Hospital Affiliated to Suzhou University, Changzhou, Jiangsu 213002, P.R. China

**Keywords:** trastuzumab, gastric cancer, sensitivity, platinum, telomere-related protein

## Abstract

*HER2* amplification occurs in ~20% of gastric cancer (GC) cases; however, in gastric and gastroesophageal junction cancer with *HER2* gene amplification, trastuzumab in combination with cisplatin (DDP)-based chemotherapy has been reported to improve the oncological outcome. The aim of the present study was to evaluate the combined antitumor efficacy of trastuzumab and various platinum agents in GC cells and to elucidate mechanisms that may be involved in the interaction between trastuzumab and the platinum agents. The *in vitro* chemosensitivity of the GC cells to platinum agents was evaluated using the CellTiter 96^®^ AQueous One Solution Cell Proliferation Assay kit. Treatment with 1.0μg/ml trastuzumab for 48 h significantly increased the sensitivity of NCI-N87 cells with *HER2* amplification to oxaliplatin (Oxa) and DDP. This chemosensitivity was most prominent in the NCI-N87 cells, in which the half maximal inhibitory concentration of Oxa and DDP was decreased to ~3.29 and 6.91 times, respectively. The apoptotic effect of the platinum agents was evaluated by double-staining the GC cells with Annexin V-fluorescein isothiocyanate and propodium iodide. Consistent with the chemosensitivity analysis, apoptotic analysis indicated that trastuzumab significantly increased Oxa- and DDP-induced apoptosis in the NCI-N87 cells. Furthermore, the mRNA expression levels of various telomere-associated genes was determined by performing quantitative reverse transcription-polymerase chain reactions in a number of GC cell lines, and revealed that trastuzumab (alone and in combination with DDP) may downregulate the mRNA expression levels of the *TPP1*, *TRF1*, *TRF2*, *TRF2IP* and *POT1* genes. However, western blot analysis demonstrated that trastuzumab (alone and in combination with DDP) may significantly downregulate the protein expression levels of telomeric repeat binding factor 2, protection of telomere 1 and TPP1 (formerly known as TINT1, PTOP and PIP). The results of the present study indicate a potential role of low-dose trastuzumab administration for increasing Oxa and DDP sensitivity in *HER2*-amplified GC cells, possibly via the downregulation of telomere-associated gene expression.

## Introduction

Globally, gastric cancer (GC) is the predominant cause of gastrointestinal cancer and is the second leading cause of cancer-related mortality (1,2). Despite the use of curative resection for the treatment of resectable GC, recurrence rates remain high following surgery. Furthermore, the majority of patients are diagnosed at an advanced stage of GC, at which surgical resection is no longer feasible; although palliative radiotherapy and chemotherapy provide some benefit, the prognosis of advanced GC remains poor (3–6).

Various attempts have been made to improve the objective response rate to GC treatment, including the development of biologically targeted agents in combination with traditional chemotherapy regimens; for example, trastuzumab in combination with a cisplatin (DDP)-based chemotherapy regimen. Trastuzumab is a humanized monoclonal anti-human epidermal growth factor receptor 2 (HER2) antibody treatment. The first phase III, prospective, randomized, multicenter trial to evaluate its efficacy and safety for HER2-positive GC treatment was the Trastuzumab for Gastric Cancer (ToGA) study (7–9). Although trastuzumab (Herceptin™) in combination with a DDP-based chemotherapy regimen produced a significant overall survival benefit, the benefit was modest and the mechanism involved was not clearly addressed.

A number of *in vitro* and *in vivo* studies have demonstrated that the administration of trastuzumab in combination with chemotherapeutic agents produces an additive effect, a synergistic effect or both in breast cancer (10–13). Furthermore, previous *in vitro* studies demonstrated that trastuzumab in combination with DDP (14) and doxorubicin (15) produces a synergistic effect in human *HER2*-overexpressing GC cells; however, the mechanisms of this synergistic anticancer activity are yet to be fully explored.

Chromosome ends consist of specialized structures called telomeres that are critical to chromosome integrity. In vertebrates, telomeres consists of thousands of T2AG3 hexamer repeats; the nucleophilic sites of these repeats are able to react with platinum agents to form DNA adducts, principally at adjacent deoxyguanines (GpG). The DNA adducts formed are 1,2-intrastrand cross-links, which result in the efficient inhibition of DNA replication, RNA transcription, cell cycle arrest or apoptosis. Subject to the presence of two or more tandem guanines, platinum agents exhibit maximal targeting of the DNA; thus, telomeric repeats are a good target for platinum agents (16). Furthermore, a previous *in vitro* study demonstrated that telomere dysfunction may increase DDP sensitivity in melanoma cells (17) and an increasing number of proteins have been discovered to interact with telomere DNA repeats; for example, telomere protection, function, and length appear to depend on the shelterin protein complex [telomeric repeat binding factor 1 (TRF1), TRF2, TPP1 (formerly known as TINT1, PTOP and PIP), protection of telomere 1 (POT1), TRF1-interacting nuclear factor (TIN2), and TRF2-interacting protein 1 (TRF2IP)] (18). The ToGA study indicated that trastuzumab in combination with a DDP-based chemotherapy regimen resulted in a significant overall survival benefit (9). In the present study, we hypothesize that trastuzumab may additionally affect the expression levels of the abovementioned telomere-associated proteins and, thus, the sensitivity of GC cells to platinum agents.

The present preclinical study was undertaken to investigate the effect of low-dose trastuzumab on the sensitivity of GC cells to platinum agents, and to elucidate the possible mechanisms involved in the interaction between the trastuzumab and platinum agents. In addition, the protein and mRNA expression levels of telomere-associated genes and proteins was investigated in GC cells following treatment with trastuzumab and platinum agents, alone and in combination.

## Methods

### Cell lines and cell culture

The effects of trastuzumab, Oxa, DDP, 5-fluorouracil (FU) and taxol administration (alone and in combination) on malignant cell growth were studied in a panel of five human GC cell lines (AGS, NCI-N87, MGC-803, HGC and MKN45) obtained from the Shanghai Institute of Cell Biology (Shanghai, China). Of these, NCI-N87 is an *HER2*-amplified cell line (19). All of the cell lines were cultured in RPMI-1640 medium (Gibco Life Technologies, Carlsbad, CA, USA) supplemented with 10% bovine serum (Invitrogen Life Technologies, Carlsbad, CA, USA), 100 mg/ml streptomycin (Sichuan Pharmaceutical Co., Ltd., Sichuan, China), 100 U/ml penicillin (Sichuan Pharmaceutical Co., Ltd.), insulin (Tonghua Dongbao Pharmaceutical Co., Ltd., Jilin, China), glutamine and pyruvate (Invitrogen Life Technologies) at 37°C in a 5% CO_2_ water-saturated atmosphere.

### Platinum agents

Prior to each experiment, the dilutions of all of the reagents were freshly prepared. Trastuzumab was obtained from the University of California Pharmaceutical Services (Los Angeles, CA, USA) and was prepared from a stock concentration of 20 mg/ml. Oxa, DDP, 5-FU and Taxol were supplied by the Jiangsu Hengrui Medicine Co., Ltd. (Lianyungang, China). A CellTiter 96^®^ AQueous One Solution Cell Proliferation Assay kit was purchased from Promega Corporation (Madison, WI, USA) and the Annexin-V-fluorescein isothiocyanate (FITC) Apoptosis Detection kit was purchased from Invitrogen Life Technologies.

### Cell viability assay

The CellTiter 96 kit (Promega Corporation) was used to determine cytotoxicity, according to the manufacturer’s instructions. Briefly, the five human GC cell lines were grown to the log phase, trypsinized, seeded into 96-well plates at a density of 2×10^3^ cells/well and incubated overnight to allow cell adherence. Subsequently, the medium in each well was replaced with fresh (platinum agent-free) medium or medium containing various concentrations (1×10^−4^, 1×10^−3^, 1×10^−2^, 1×10^−1^, 1, 10 and 100 μg/ml) of platinum agents (Oxa and DDP) and was incubated for an additional 48 h. CellTiter 96 AQueous One solution was added to each well at one fifth of the mixture volume and the plates were incubated for 3 h. Absorbance was determined at an wavelength of 490 nm using a microplate reader (Bio-Rad Laboratories, Hercules, CA, USA), with blank control wells to zero the absorbance. For each experiment, 10 control wells were allocated for platinum agent-free medium and a minimum of six replicate wells were allocated for each concentration of platinum agent-containing medium. Using the background-corrected absorbance values, the inhibition rate [I (%)] was calculated by the following equation: I (%) = 100 × (A_untreated control well_ − A_experimental well_)/A_untreated control well_. The half maximal inhibitory concentration (IC_50_) was defined as the concentration of platinum agent required for 50% inhibition of cell growth.

### Apoptosis assay

The number of apoptotic cells was quantified using an Annexin V-FITC Apoptosis Detection kit (Invitrogen Life Technologies), according to the manufacturer’s instructions. Briefly, the NCI-N87 cells were grown to 75–80% confluence in 60-mm Petri dishes, exposed to trastuzumab (1.0 μg/ml) and platinum agents (Oxa, 5 μg/ml; DDP, 2.5 μg/ml) alone or in combination for 48 h, and compared with the untreated control cells. To quantify the apoptosis, the cells were collected, resuspended in 500 μl binding buffer, treated with 5 μl Annexin V-FITC and 5 μl propidium iodide (PI), and analyzed using a FACSCalibur™ flow cytometer (BD Biosciences, Franklin Lakes, NJ, USA).

### Reverse transcription-quantitative polymerase chain reaction (RT-qPCR)

Briefly, the three human GC cell lines, NCI-N87, HGC27 and MKN45, were grown to the log phase, trypsinized, washed with phosphate-buffered saline (PBS) and collected by performing centrifugation for 5 min at 174 × g. Total RNA was extracted from each cell line using the SV Total RNA isolation system (Promega Corporation), according to the manufacturer’s instructions. The purity and quality of the extracted mRNA were determined using a Bio-visible spectrophotometer at 260 and 280 nm (Eppendorf, Hamburg, Germany); and the integrity of the extracted mRNA was determined by performing agarose gel electrophoresis on a 1% gel. Reverse transcriptase from the reverse transcription system was used according to the manufacturer’s instructions (Promega Corporation) to synthesize a total volume of 20 μl complementary DNA for each cell line, and the iCycler iQ™ Multi-Color Real Time PCR detection system (Bio-Rad Laboratories, Inc.) was used to perform RT-qPCR of the target genes and the internal control (*β-actin*). Applied Biosystems Life Technologies (Foster city, CA, USA) supplied the primers [1X; Assay IDs: Hs00819517_mH (*TRF1*); Hs01554305_g1 (*TIN2*); Hs00194619_m1 (*TRF2*); Hs00368526_g1 (*TPP1*); Hs00430292_m1 (*TRF2IP*); Hs00209984_m1 (*POT1*); and Hs99999903_m1 (*β-actin*)] and probe mixture, and AbGene Ltd. (Surrey, UK) supplied the ABsolute qPCR mix (1X) used to produce the 20-μl PCR reaction mixture. The PCR conditions were as follows: 50°C for 2 min, 95°C for 15 min, followed by 45 cycles at 95°C for 15 sec and 60°C for 1 min. Using β-actin as an endogenous control and commercial human total RNA samples (Clontech Laboratories, Inc., Mountainview, CA, USA) as calibrators, the relative gene expression levels were quantified according to the comparative Ct method, employed the formula 2^−ΔΔCt^ to determine the final results (20). Following PCR, the 10-μl product was loaded onto a 1.5% agarose gel and visualized by ethidium bromide staining (Sigma-Aldrich, Munich, Germany).

### Western blot analysis

Total cell lysates were prepared in RIPA lysis buffer (Pierce Biotechnology, Inc., Rockford, IL, USA). Following determination of protein concentration using a bicinchoninic acid protein assay kit (Pierce Biotechnology, Inc.), an aliquot of lysate containing 50 μg of each protein was subjected to sodium dodecyl sulfate-polyacrylamide gel electrophoresis, transferred to polyvinylidene fluoride membranes, blocked with blocking buffer (PBS Tween-20 containing 5% non-fat milk) for 2 h at room temperature and incubated overnight at 4°C with the following specific primary antibodies: monoclonal mouse anti-human TPP1 (ACD; cat. no. TA504406), monoclonal rabbit anti-human POT1 (cat. no. TA310771), polyclonal rabbit anti-human TRF1 (cat. no. TA322887), rabbit anti-human monoclonal TRF2 (cat. no. TA307200), rabbit anti-human polyclonal TIN2 (cat. no. TA315321), rabbit anti-human polyclonal TRF2IP (cat. no. TA324532 ) and rabbit anti-human polyclonal GAPDH (cat. no. TA308884) (1:10,000, all primary antibodies; OriGene Technologies, Inc., Rockville, MD, USA). Subsequent incubation with the appropriate horseradish peroxidase-conjugated goat anti-rabbit POT1, TRF1, TRF2, TIN2, TRF2IP, GAPDH (cat. no. SP 9001) and goat anti-mouse TPP1 (cat. no. SP9002) secondary antibodies (1:1,000; Santa Cruz Biotechnology, Inc., Santa Cruz, CA, USA) was performed for 2 h at room temperature. Signals were then detected using enhanced chemiluminescence reagents (Thermo Fisher Scientific, Waltham, MA, USA), and a FluorChem SP imaging system (Alpha Innotech, San Leandro, CA, USA) was used for image capture.

### Statistical analysis

Values are expressed as the mean ± standard deviation. All statistical analyses were performed using SPSS version 13.0 software (SPSS, Inc., Chicago, IL, USA). Statistical comparison was performed using Student’s t-test and P<0.05 was considered to indicate a statistically significant difference.

## Results

### Trastuzumab renders HER2-amplified cancer cells sensitive to platinum agents

**Figure 1:**
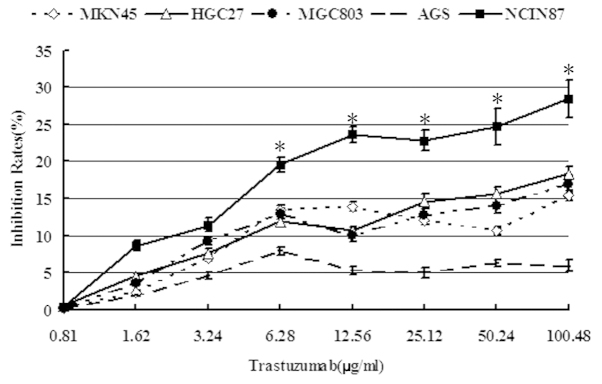
Trastuzumab caused significantly more cytotoxicity to the NCI-N87 cell line with *HER2* amplification compared with the other four gastric cancer cell lines investigated. ^*^P<0.05 vs. MKN45, HGC27, MGC-803 and AGS cell lines.

The cytotoxicity of trastuzumab to five GC cell lines was initially analyzed using a CellTiter 96 Aqueous One Solution Cell Proliferation Assay kit. It was identified that trastuzumab (6.28, 12.56, 25.12, 50.24 and 100.48 μg/ml)rendered more significant cytotoxicity to the NCI-N87 cell line with *HER2* amplification compared with the four other cell lines (P<0.05). At 0.81–100.48 μg/ml trastuzumab, the inhibition rates to NCI-N87 cells were not >30% and 0.81–1.62 μg/ml trastuzumab was not obviously cytotoxic to cancer cells (survival rate, >90%; Fig. 1). Thus, as treatment of the cells with 1.0 μg/ml trastuzumab exhibited no significant effect on cell viability, this concentration was used for subsequent analyses.

Additionally, 1.0 μg/ml trastuzumab was administered to five GC cell lines and the effect on the sensitivity of the cell lines to various platinum agents was investigated. It was identified that pretreatment with trastuzumab significantly increased the sensitivity of only the NCI-N87 cell line to platinum agents. As indicated in Table I, the IC_50_ of Oxa and DDP was decreased to ~3.29 (P=0.001) and 6.91 times (P=0.002) in NCI-N87 cells, respectively; however, trastuzumab did not alter the IC_50_ of Oxa or DDP in the other four cell lines. Subsequently, it was identified that simultaneous treatment with low-dose trastuzumab and platinum agents may significantly increase the sensitivity of NCI-N87 cells to platinum agents; for example, the IC_50_ of Oxa and DDP were reduced by ~2.67 (P=0.001) and 4.56 times (P=0.003), respectively. Thus, these data indicate that low-dose trastuzumab may increase platinum sensitivity in NCI-N87 cells.

### Trastuzumab treatment increases platinum agent-induced apoptosis in NCI-N87 cells

**Figure 2:**
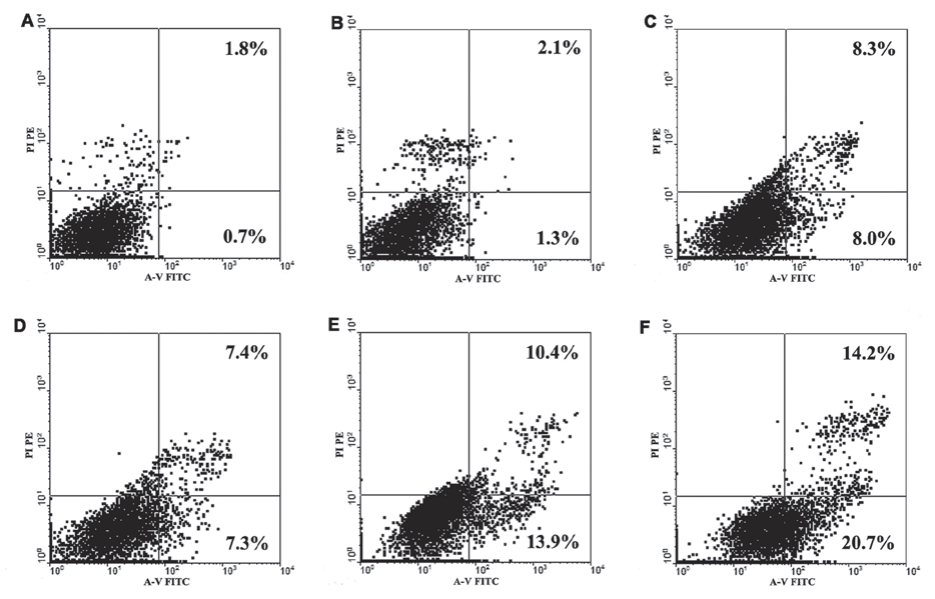
Trastuzumab treatment increases oxaliplatin- and cisplatin-induced apoptosis in NCI-N87 cells. (A) Untreated cells, and cells treated with (B) trastuzumab alone, (C) oxaliplatin alone, (D) cisplatin alone, (E) trastuzumab in combination with oxaliplatin and (F) trastuzumab in combination with cisplatin. A-V FITC, Annexin-V fluorescein isothiocyanate; PI PE, propidium iodide phycoerythrin.

To further investigate whether low-dose trastuzumab increases the sensitivity of NCI-N87 cells to platinum agents, GC cells were double-stained with Annexin V-FITC and PI to detect early apoptosis induced by treatment with platinum agents and trastuzumab, alone or in combination. The doses of Oxa and DDP selected were 5 μg/ml and 2.5 μg/ml, respectively, as they were close to the 20% inhibitory concentrations (IC_20_) of NCI-N87 cells. The dose of trastuzumab used was 1.0 μg/ml, as previously determined. For the groups treated with two agents in combination, the cells were pretreated with trastuzumab for 48 h, followed by the addition of platinum agents for 48 h. As indicated in Fig. 2, the percentage of early apoptosis induced by independent Oxa and DDP administration in NCI-N87 cells was 8.0 and 7.3%, respectively; however, this increased to 13.9 and 20.7% upon treatment with trastuzumab in combination with Oxa and DDP, respectively. Furthermore, treatment with trastuzumab alone (1.0 μg/ml) did not induce significant apoptosis in the cancer cells. Thus, it appears that low-dose trastuzumab administration may increase platinum agent-induced apoptosis in NCI-N87 cells and subsequently result in the increased cytotoxicity of platinum agents (Fig. 1).

### Trastuzumab downregulates mRNA and protein expression levels of telomere-associated genes in NCI-N87 cells

**Figure 3:**
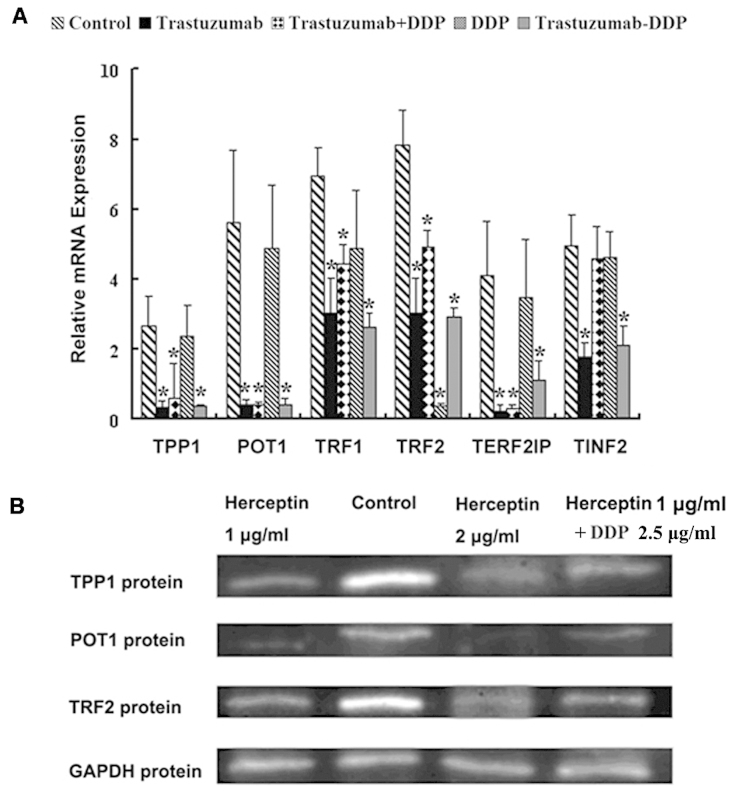
Effect of trastuzumab administration on the expression of telomere-associated genes and proteins in NCI-N87 cells. (A) Reverse transcription-quantitative polymerase chain reaction was used to determine the levels of *TPP1*, *POT1*, *TRF1*, *TRF2*, *TERF2IP* and *TINF2* mRNA expression in NCI-N87 cells treated with trastuzumab and cisplatin (alone or in combination). Error bars indicate the standard deviation from the mean. ^*^P<0.05, vs. control cells. (B) Western blot analysis determined that the protein expression levels of TPP1, POT1 and TRF2 in NCI-N87 cells were significantly inhibited by various concentrations of trastuzumab alone and low-dose trastuzumab in combination with cisplatin. TPP1 (formerly known as TINT1, PTOP and PIP); POT1, protection of telomere 1; TRF2, telomeric repeat binding factor 2; Control, untreated NCI-N87 cells.

To understand the mechanisms by which trastuzumab may increase the sensitivity of NCI-N87 cells to platinum agents, we hypothesized that trastuzumab may affect the expression levels of telomere-associated genes or proteins in NCI-N87 cells, thus, influencing their sensitivity to platinum agents. Following incubation with 1.0 μg/ml trastuzumab alone, 2.5 μg/ml DDP alone or the two agents in combination, the mRNA expression of various telomere-associated genes and their proteins were assessed in NCI-N87 cells by performing RT-qPCR and western blot analysis. As demonstrated in Fig. 3A, the mRNA expression levels of *TRF1*, *TRF2*, *POT1*, *TPP1* and *TRF2IP* were significantly downregulated following treatment with trastuzumab alone or DDP in combination with trastuzumab in NCI-N87 cells (P<0.05), and DDP alone only downregulated the expression level of *TRF2* (P<0.05). However, in the HGC27 and MKN45 cell lines, treatment wth low dose trastuzumab alone or in combination with DDP did not significantly downregulate the mRNA expression levels of any of the telomere-associated genes (P>0.05) (data not shown). Western blot analysis indicated that treatment with trastuzumab alone and in combination with DDP significantly downregulated the protein expression levels of TRF2, POT1 and TPP1 in NCI-N87 cells, and DDP alone did not downregulate the protein expression level of any of the telomere-associated genes investigated (data not shown). The results indicated that low-dose trastuzumab administration may downregulate the mRNA and protein expression levels in an area of the telomere-associated genes that may be involved in chemosensitivity NCI-N87 cells to platinum agents.

## Discussion

Overexpression and activation of *HER2* has previously been associated with chemotherapy resistance in *HER2* gene-amplified cancer cells (21–23), and telomere dysfunction has been associated with platinum sensitivity in cancer cells (17). The aim of the present study was to investigate whether the antitumor activity of platinum agents commonly used in the treatment GC could be enhanced by the addition of low-dose trastuzumab and to discuss the possible mechanisms of platinum agent-resistance in GC cells, which may involve telomere-associated proteins. First, it was investigated whether low-dose trastuzumab could increase the sensitivity of five GC cell lines to platinum agents by performing cell proliferation and early apoptosis analysis. The findings of the present study indicate that treatment with low-dose trastuzumab may reduce the IC_50_ of platinum agents and increase the early apoptosis rates induced by platinum agents in NCI-N87 cells, which overexpress *HER2* gene; however, this increase in sensitivity was not observed in the other four cell lines. Additionally, the expression levels of telomere-associated genes appeared to be downregulated in NCI-N87 cells following treatment with low-dose trastuzumab alone or trastuzumab in combination with DDP. These results indicate that pretreatment with low-dose trastuzumab followed by platinum agent administration may be a promising method the treatment of HER2-positive advanced GC. Thus, a possible, partial mechanism for the increase in platinum agent sensitivity may be the downregulation of the expression of telomere-related genes.

HER-2/neu or c-erbB-2 is a member of the HER family of growth factors (endothelial growth factor receptor, erbB-2, erbB-3 and erbB-4), which exhibit intrinsic protein tyrosine kinase activity. Increased HER-2 activity is the assumed mechanism underlying cell cycle control, proliferation, differentiation, motility, apoptosis, metastasis and transformation (14,24–26) and *HER-2* overexpression is observed in numerous human carcinomas, including breast, ovarian, gastric, colon and non-small cell lung cancer. Trastuzumab, a humanized monoclonal antibody (mAb) against the extracellular domain of HER2, has been approved by the Food and Drug Administration for the treatment of patients with invasive *HER2*-overexpressing breast cancer (27). Additionally, *HER2*-amplified GC is the most probable candidate for responding to trastuzumab treatment, as various studies have demonstrated that an antitumor effect occurs when trastuzumab is added to *HER2*-amplified GC cells or the corresponding xenograft models (19,28,29). Additionally, the results of the present study demonstrated significant cytotoxicity of trastuzumab on the *HER2*-amplified NCI-N87 cell line; however, the inhibition rates were not >30%, even at the highest concentration. These results are consistent with those of a previous *in vitro* study (30), which indicated that the administration of trastuzumab alone may not exhibit a significant antitumor effect.

Numerous *in vitro* and *in vivo* studies have demonstrated that additive and synergistic effects occur when trastuzumab is combined with other chemotherapeutic agents to treat breast cancer and GC (10–14). The mechanisms by which this occurs include a reduction in DNA repair activity following chemotherapeutic agent-induced DNA damage (31,32), inhibition of the unscheduled DNA synthesis (33), and increased apoptosis via the activation of antibody responses and reduced expression of anti-apoptotic genes (34,35).

In the present study, it was identified that low-dose trastuzumab administration may significantly decrease the IC_50_ of platinum agents and increase the early apoptosis rates induced by them in NCI-N87 cells, which overexpress the *HER2* gene, but not in other four cell lines. These results indicated that the HER2/neu signaling pathway may participate in the mechanisms of platinum agent chemosensitivity in *HER2-*amplified GC. The results obtained in the present study are partially consistent with those reported by Yu and Hung (36), Funato *et al* (37), and Järvinen and Liu (38). For example, Yu and Hung (36) reported that the trastuzumab-induced increase in paclitaxel sensitivity in *HER2*-overexpressing breast cancer cells may occur by reversing the anti-apoptotic function of HER2. In the present study, the administration of trastuzumab alone at 1.0 μg/ml did not induce significant apoptosis in the NCI-N87 cells; however, when the NCI-N87 cells were treated with trastuzumab in combination with DDP or Oxa for 48 h, the early apoptotic rates were markedly increased to 20.7 and 13.9%, respectively. Thus, it appears that trastuzumab may increase platinum agent-induced apoptosis in NCI-N87 cells, resulting in the increased cytotoxicity of platinum agents.

Telomeres are nucleoprotein complexes located at the ends of chromosomes. In vertebrates, telomeres consists of tandem repeats of the T2AG3 hexamer, a G-rich motif and associated proteins (39). Due to the presence of guanine triplets, telomeric DNA is considered to be a preferential target for DDP (16). Furthermore, telomeres are critical for genomic stability as they provide a mechanism for the maintenance and protection of chromosomal ends by folding into a structure termed the T-loop. The T-loop is characterized by a single-stranded overhang of 30 nucleotides, which is sequestered by invasion of a duplex region of the telomere (39). TRF1, TRF2, POT1, TRF2IP, TPP1 and TIN2 form a six-protein complex termed shelterin (18), which contributes to the formation of the T-loop as well as the telomere protection and length regulation. Within shelterin, TRF1, TRF2 and POT1 bind directly to the telomeric DNA; TRF1 forms a complex with a number of proteins, including tankyrase, TIN2, TPP1 and POT1 (40–43); and TRF2 interacts with various proteins, including TRF2IP, TIN2 and POT1 (41,42,44–46). The uncapping of telomeres due to dysfunctional telomeric proteins or telomere shortening can disrupt their protective function and activate a DNA damage response (47,48). The loss of telomere protection, whether induced by telomere shortening or via the disruption of telomere structure, is commonly referred to as telomere dysfunction, and has been reported as the principal determinant governing chemosensitivity against agents that induce double-strand DNA breaks (49,50).

In conclusion, the present study identified that the mRNA and protein expression levels of TRF2, POT1 and TPP1 were significantly downregulated following treatment with trastuzumab alone or trastuzumab in combination with DDP. Thus, it is proposed that the HER2/neu signaling pathway modulates the expression of a number of telomere-associated proteins, resulting in telomere dysfunction. Telomere dysfunction may represent a physiological trigger of the DNA damage or apoptotic response, analogously to other genotoxic insults that introduce chromosome breaks. Concurrently, telomere dysfunction may additionally contribute to sensitizing the platinum agents in *HER2*-amplified GC cells. The mechanisms by which trastuzumab influences the expression of these telomere-associated proteins remain unclear; however, a possible mechanism may involve changes in numerous signal transduction pathways associated with drug-gene or drug-protein interactions. Thus, additional studies are required to analyze the effects of platinum agents on the gene and protein expression profiles of various signaling pathways.

## Figures and Tables

**Table I:** Effects of trastuzumab on the IC_50_ of DDP and Oxa in five gastric cancer cell lines.

	Gastric cancer cell line (IC_50_, mean ± standard deviation)				
	
Agent	NCI-N87^a^	HGC27	MGC803	AGS	MKN45
DDP	12.86±1.41	10.23±1.94	15.26±1.85	9.39±1.77	26.44±2.72
DDP (trastuzumab)^b^	1.86±0.55^c^	10.97±2.36	14.38±1.63	10.19±3.02	25.44±4.70
Oxa	21.53±1.96	15.07±3.30	27.26±3.39	19.21±1.85	35.77±3.82
Oxa (trastuzumab)^b^	6.53±1.10^d^	14.66±2.66	26.69±4.47	20.39±3.70	37.64±6.66

a Overexpressing HER2/neu gene;

b pretreatment with trastuzumab for 48 h;

c P=0.002, vs. DDP group;

d P=0.001, vs. Oxa group.

IC_50_, half maximal inhibitory concentration; DDP, cisplatin; Oxa, oxaliplatin.
